# A Systematic Review on Psychological Interventions for Sexual Health in Older Age

**DOI:** 10.1080/19317611.2023.2215766

**Published:** 2023-05-30

**Authors:** Priscila Vasconcelos, Rodolfo Gomez Ponce de Leon, Suzanne J. Serruya, Bruna Carneiro, Catarina Nóbrega, Raquel Pereira, Ana Quinta Gomes, Maria Constança Paúl, Pedro J. Nobre

**Affiliations:** a Faculty of Psychology and Education Sciences, Center for Psychology at University of Porto, Porto, Portugal; b Centro Latinoamericano de Perinatología, Salud de la Mujer y Reproductiva (CLAP-SMR/OPS-OMS), Montevideo,Uruguay; c ICBAS, University of Porto, Portugal, Porto, Portugal

**Keywords:** Psychological intervention, older age, sexuality, sexual health, evidence-based

## Abstract

**Objectives:**

The present review aims to identify the existing evidence on outcome-treatment studies of psychological sexual health interventions in older age.

**Methods:**

A systematic search was conducted for studies published until October 2022. Data search was conducted on EBSCO, MEDLINE, Web of Science, and Cochrane Central Register of Controlled Trials databases.

**Results:**

From 30,840 screened records, 12 reports were selected. Results were grouped into four categories according to the intervention that was implemented.

**Conclusions:**

Despite results presenting some bias concerns, this review suggests that educational and cognitive-behavioral approaches seem to be effective for promoting sexual health in older age.

## Introduction

According to the World Social Report 2023, “population aging is an irreversible global trend” (United Nations [UN], 2023, p. 17) that stems from low fertility rates and higher life expectancy. The promotion and maintenance of health and well-being in older age is a critical public health goal, as reflected in the Sustainable Development Goals (SDGs) and the United Nations Decade of Healthy Aging, highlighting the importance of prioritizing the health needs of the aging population (Sadana et al., 2018; UN, 2023). Global public health recognizes sexual health promotion as a significant concern. The SDGs incorporated sexual and reproductive health and rights into the UN 2030 agenda, with the premise of improving overall health across the lifespan (UN, 2015). Similarly, the World Health Organization (WHO, 2015) recognizes that sexual health is essential to personal fulfillment and well-being, regardless of age. Such developments are in accordance with the acknowledgment of sexual health as a human right (World Association for Sexual Health, 2014), with sexual pleasure emerging in this triad as a fundamental aspect of sexual health and well-being across the lifespan, including later life (Sladden et al., 2021).

Evidence on sexuality and aging indicated that as age progresses there is an increased likelihood of developing sexual dysfunctions or difficulties (Peixoto & Nobre, 2015; Quinta Gomes & Nobre, 2011; Træen et al., 2017) and having less frequent sexual activities (Gillespie, 2017; Herbenick et al., 2010). Despite the common belief that aging entails refraining from engaging in sexual activities (DeLamater, 2012; Freak-Poli et al., 2017), studies have shown that older individuals maintain their sexual interest and enjoyment in later adulthood (Freak-Poli, 2020; Træen et al., 2018). Rather than age, factors such as partner availability (T. J. Flynn & Gow, 2015; Freak-Poli et al., 2017) and health status (D. M. Lee, Nazroo, et al., 2016; Lindau et al., 2007) are important for sexual expression in later life. On the other hand, findings suggest that having a fulfilling sexual life is beneficial for health and quality of life (T. J. Flynn & Gow, 2015; Smith et al., 2019; Willie-Tyndale et al., 2021; Wright et al., 2019). Maintaining sexual desire and sexual satisfaction in older age was linked with multiple dimensions of well-being (Buczak-Stec et al., 2019; Jackson et al., 2019; D. M. Lee, Vanhoutte, et al., 2016; Smith et al., 2019) and successful aging (Štulhofer et al., 2018)., thus underlining the importance of adopting a more positive approach toward sexuality and aging, namely including sexual health and pleasure, for promoting well-being in later adulthood (Cismaru-Inescu et al., 2022; Sladden et al., 2021; Syme et al., 2019)

Studies demonstrated that psychological interventions—a set of strategies and techniques applied by trained professionals aimed at promoting positive change and alleviating distress by modifying less adaptive emotional reactions, ways of thinking, and behavioral patterns (VandenBos, 2015)—effectively diminished male and female sexual difficulties (Bergeron et al., 2021; Bilal & Abbasi, 2022; Brotto et al., 2022; Zippan et al., 2020). However, treatment-efficacy studies for sexual problems in older age remain sparse. A comprehensive review on the management of sexual dysfunctions in older age performed by Fisher and colleagues found only one non-pharmacological treatment-efficacy study (Fisher et al., 1997). Even though there were several interventional guidelines, most of the literature on promoting sexual health in older age proposed interventions without empirical evidence of their effectiveness (e.g. Fazio, 1988; Lee et al., 2012).

Considering the latest developments in public health goals that underscore the importance of prioritizing sexual health and well-being in older age, the present systematic review aims at identifying psychological interventions with tested effectiveness amongst older adults.

## Methods

### Data search

This systematic review followed the guidelines from Preferred Reporting Items for Systematic Reviews (PRISMA) (Page et al., 2021). No pre-registration was performed.

The literature search was conducted in October 2022 using EBSCO (APA PsycARTICLES, APA PsycINFO), MEDLINE (PubMed) Web of Science, and Cochrane Central Register of Controlled Trials. Different combinations of terms were used aiming at increasing the number of results concerning sexual health interventions directed to older adults. The search strategy was widened to detect medical or pharmaceutical interventions that potentially included a psychological component. Specifically, we conducted the search using the following string: (intervention OR program OR treatment OR therapy) AND (“sexual health” OR sexuality OR “sexual wellbeing”) AND (aging OR aging OR elderly OR older adults OR seniors OR geriatrics).

### Eligibility criteria

For the screening purpose, inclusion criteria included: (a) a study sample whose mean age was 50 years or older; (b) the description of the implementation of a psychological intervention directed to older people; (c) the quantitative assessment of sexual health outcomes (e.g. sexual knowledge, sexual attitudes, sexual function, sexual behavior, sexual satisfaction); and (d) the implementation of analytical methods to test the effect of the intervention described in the study. Exclusion criteria included: (a) participants presenting significant physical or mental health issues (e.g., neoplasia, neurodegenerative pathologies, cardiovascular disease); (b) review study on sexuality and aging or sexual health intervention; (c) absence of the quantitative data required to assess intervention effects; and (d) not being a published original study written in English. Gray literature was also excluded. No data restriction was applied.

### Study selection and data extraction

After the identification of the studies and duplicates removal, search results were transferred to Rayyan database. PV performed the screening based on title, abstract, and full text to select the records that fulfilled the inclusion criteria. The remaining authors solved potential inclusion disagreements. PV conducted the risk of bias assessment. Rating disagreements of risk of bias were solved by non-blinded consensus between all authors.

Relevant data for the qualitative synthesis of the results were obtained from the studies that met the eligibility criteria. In particular, studies were coded according to the name of the first author, year of publication, the country where the intervention was implemented, sample characteristics, intervention and setting (i.e. type of intervention and delivery format), frequency and length of sessions, outcome measures, analytical approach, and outcome results.

Each intervention described in the included studies was classified based on the pre-specified categorization of interventions for sexual dysfunctions proposed by Frühauf et al. (2013): (a) Cognitive-behavioral therapy (CBT), that is, interventions aimed at changing erroneous beliefs and patterns of thinking (cognitive restructuring), with homework assignments, out-of-session activities, psychoeducation, and skills building being emphasized in this treatment; (b) Sex therapy (ST), that is, interventions based on Masters and Johnson’s (1970) intervention program for sexual dysfunction that includes psychoeducation, couple exercises (e.g., sensate focus exercises), and counseling. This category includes not only therapies outlined by Masters and Johnson’s (1970) proposal but also modified versions of sexual therapy; (c) Sexual skills training (SST), that is, interventions based on providing a set of exercises aimed at assisting participants in enhancing sexual functioning techniques. Masturbation training, the stop-and-start technique, and other exercises may be performed individually and/or with the partner; (d) Educational intervention (EI), that is, interventions that are mainly focused on conveying information regarding the changes that might occur in sexual health as age progresses; and (f) Other psychotherapy (OP), for example, emotion regulation therapy. For each intervention, the treatment setting was classified according to the following categories: individual, couple, or group setting. For the 9 randomized studies included in this review, the risk of bias was assessed by implementing the Revised Cochrane risk-of-bias tool for randomized trials (Sterne et al., 2019).

## Results

**Figure 1.**
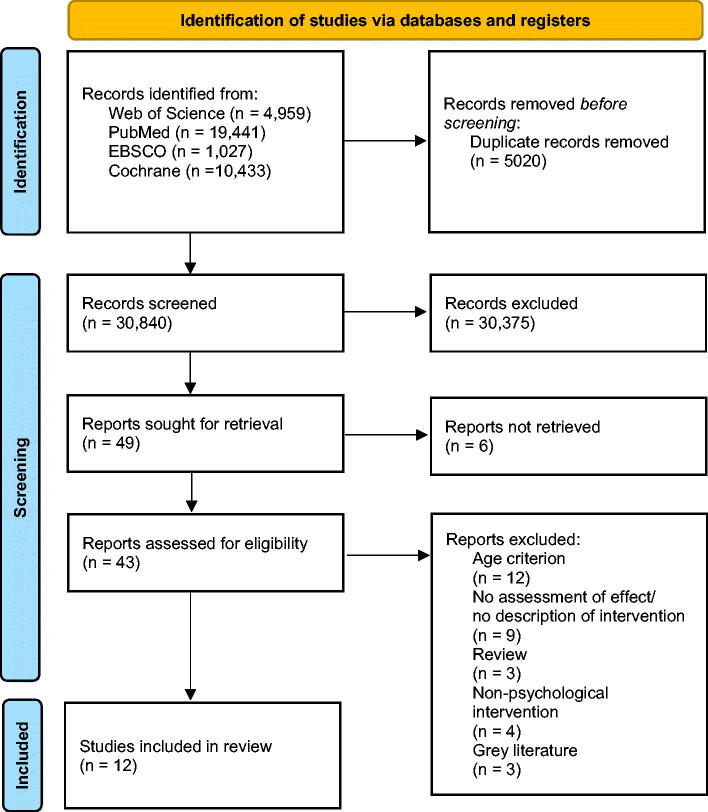
PRISMA Flowchart of data screening and study selection.

The literature search yielded a total of 35,860 entries. After duplicate removal, 30,840 records were screened based on title, 465 records were screened based on abstract and 43 reports were assessed for eligibility. The full-text screening of potentially relevant articles assessed against the eligibility criteria resulted in a total of 12 studies. Figure 1 illustrates the process of data screening and study selection.

**Figure 2.**
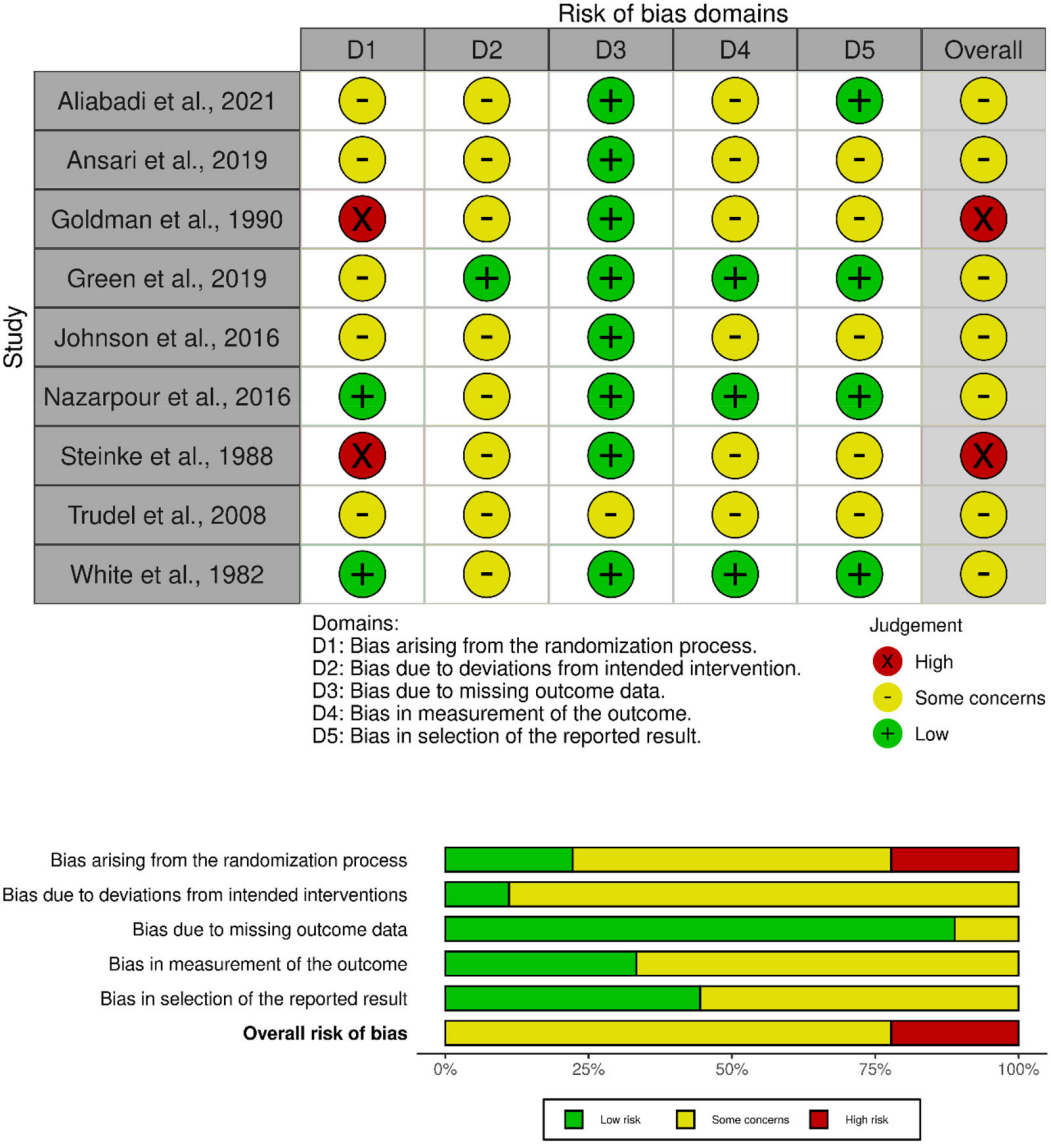
Risk of bias traffic-light plots and summary plot.

Table 1 provides information about the selected studies. In summary, five studies performed comparisons reporting on aging sexual knowledge and sexual attitudes, and on sexual satisfaction or well-being, five studies conducted comparisons between pre and post intervention scores on sexual function and behavior, one study each assessed the effect of the intervention on sexual fantasies and on sexual quality of life. The sample size ranged from 10 to 187 participants per study. The studies were published between 1978 and 2021. Six studies were conducted in the USA, three in Iran, two in Canada, and one in Switzerland. The age of the participants ranged from 39 to 91 years. Educational intervention was implemented in 5 studies, followed by cognitive-behavioral therapy (*n* = 3), marital therapy (*n* = 2), sexual therapy (*n* = 2), sexual skills training (*n* = 1), and other psychotherapy (*n* = 3). From all interventions included in this review, 8 were conducted in a group setting, three were performed in a couple setting and one was conducted in an individual setting. Within the 12 studies included in this review, 5 studies used as sexual health outcome measures the Aging Sexuality Knowledge and Attitudes Scale (White, 1982), 2 studies used the Sexual Interaction Inventory (LoPiccolo & Steger, 1974), as well as 2 studies used the Female Sexual Function Index (Rosen et al., 2000). Amongst other outcome measures were the Derogatis Sexual Functioning Inventory (Derogatis & Melisaratos, 1979), the Frequency of Sexual Behavior Form (Petzold, 1982), the Index of Sexual Satisfaction (Hudson et al., 1981), the McCoy Female Sexuality Questionnaire (McCoy, 2000), the Menopause-Specific Quality of Life Questionnaire (Hilditch et al., 1996), the sex-specific item from Greene Climacteric Scale (Greene, 1976), Sexual Activity Questionnaire (Thirlaway et al., 1996). the Sexual Behavior Questionnaire (White & Doyle, 1979), the Sexual History Inventory (Schover et al., 1982), the Sexual Desire Scale (Tremblay & Roussy, 2000), and the Sexual Desire Questionnaire (Drouin et al., 2003). Ad-hoc measures were also used to assess the effect of the interventions on sexual satisfaction and sexual fantasies. Assessment of the risk of bias indicated that seven studies presented some bias concerns and two studies showed a “high” overall risk of bias. No study qualified as having a “low” risk of bias. Results from the risk of bias assessment can be found in Figure 2 (McGuinness & Higgins, 2021).

**Table 1.** Summary of psychological sexual health interventions in older age.

Study First author (publication year) Country	Sample (*N* analyzed) Age: *M* ± *SD* (range) Sex	Intervention and setting	Frequency and length of sessions	Outcome measures	Study design	Outcome results
Adams (1990), USA	*N* = 10 Age: 61–91; men and women	(1) EI; group	(1) Two 90-min workshop	(1) ASKAS	Cohort (one group pre + post (before and after)	(1) n.s.
Alder (2006), Switzerland	*N* = 30 Age: 52.3 **±** 5.1; peri and postmenopausal women	(1) Cognitive-behavioral group therap0; group	(1) 90-min sessions; total 7 sessions	(1) MRS (2) MFSQ	Interrupted time series	(1) *F*(1, 29) = 4.2, *p* = .05 (2) *F*(1, 29) = 6.2, *p* < .05
Aliabadi (2021), Iran	*N* = 60 Age: 52.9 **±** 3.3; postmenopausal women	OP; group	(1) 120-min sessions; total 8 sessions	(1) MENQOL	Controlled clinical trial (CCT)	(1) *p* < .001
Ansari (2019), Iran	*N* = 40 Age_control_: 54.4 ± 4.79 Age_intervention:_ 57.95 ± 6.32 postmenopausal women	(1) OP; group	(1) 90-min sessions; total 8 sessions	(1) ISS	Randomized controlled trial (RCT)	(1) *p* < .05
Goldman (1990), USA	*N* = 40 Age: 55–70; 10 experimental couples with ED; 10 control couples with ED	(1) EI; couples	(1) One 4-h workshop	(1) ASKAS (2) SII (3) FSBF	Controlled clinical trial (CCT)	(1) *t*(39) = −4.06, *p* <.001 *t*(39) = 1.37, ns (2) *t*(38) = 3.63, *p <*.01 (3) *t*(39) = 1.72, *p* >.05
Green (2019), Canada	*N* = 72 Age: 53.08 ± 4.02 (43–62) peri and postmenopausal women	(1) Cognitive-behavioral group therapy (CBT); group	(1) 120-min sessions; total 12 sessions	(1) FSFI (2) GCS-sex	Randomized controlled trial (RCT)	(1) *F(*1, 52) = .80, ns (2) *F(*1, 67) = 5.20, *p* < .05
Johnson (2016), USA	*N* = 187 Age: 54.6 ± 6.8 (39–75) postmenopausal women	(1) OP; individual	(1) Five weekly sessions	(1) SAQ	Controlled clinical trial (CCT)	*F*(1, 90) = 14.87, *p* < .001 *F*(1, 90) = 7.85, *p* < .01
Nazarpour (2016), Iran	*N* = 98 Age_control_: 52.8 ± 4.0 Age_intervention_: 51.5 ± 3.4 (40–60) postmenopausal women	(1) EI; group	(1) 120-min session; group	(1) FSFI	Randomized controlled trial (RCT)	(1) FSFI(arousal domain): *p <*.01 (2) FSFI(pain domain): *p <*.05 (3) FSFI(total score): *p <*.05
Rowland (1978), USA	*N* = 20 Age: 59.8 (51–71) 10 heterosexual couples	(1) ST; (2) SST; couples	(1,2) Total 8 sessions;	(1) SII	Cohort (one group pre + post (before and after)	(1) *F*(3, 21) = 7.90, *p* < .01
Steinke (1988), USA	*N* = 24 Age: 68.8 (60–80)	(1) EI; groups	(1) 60-min sessions; total 4 sessions	(1) ASKAS (2) Frequency of sexual behavior (3) Sexual satisfaction	Controlled clinical trial (CCT)	(1) *t*(11) = 2.91, *p <*.05 (2) *t*(10) = −.94, ns (3) *t*(11) = 1.08, ns
Trudel (2008), Canada	*N* = 154 Age: 55–69 77 heterosexual couples	(1) CBT (2) ST; couple	(1,2) Total 12 sessions	(1) ASKAS (2) SHI (3) DSFI (4) SDS (5) SDQ	Controlled clinical trial (CCT)	(1) *F*(3, 78) = 12.38 , *p* < .001 (2) *F*(1, 80) = 9.98, *p* < .01 (3) *F*(12, 69) = 6.17 , *p* < .001 (4) *F*(2, 79) = 10.79 , *p* < .001 (5) *F*(1, 80) = 55.82 , *p* < .001 *F*(2, 79) = 25.08 , *p* < .001
White (1982), USA	*N* = 30 Age_control_: 69.3 Age_intervention:_ 67.8 Community-dwelling older adults	(1) EI, group	(1) Total 3 sessions	(1) ASKAS (2) Sexual fantasies (3) Sexual satisfaction (4) SBQ	Randomized controlled trial (RCT)	(1) *F*(2,27) = 32.66, *p* < .001, *R*^2^ = .71 *F*(2,27) = 23.33, *p* < .001, *R*^2^ = .63 (2) *F*(2,27) = 1.60, ns (3) *F*(2,27) =17.92, *p* < .001, *R*^2^ = .57 (4) *F*(2,27) = 5.43, *p* < .01, *R*^2^ = .29 *F*(2,27) = 12.41, *p* < .001, *R*^2^ = .48

*Notes. Measures*: ASKAS: Aging Sexuality Knowledge and Attitudes Scale; DAS: Dyadic Adjustment Scale; DSFI: Derogatis Sexual Functioning Inventory; EQ-i: Bar-On Emotional Quotient Inventory; FSBF: Frequency of Sexual Behavior Form; FSFI: Female Sexual Function Index; FSDS-R: Female Sexual Distress Scale–Revised; GCSsex: Domain of Loss of Interest in Sex from the Greene Climacteric Scale; HADS: Hospital Anxiety and Depression Scale; ICQ: Interpersonal Communication Questionnaire; ISS: Index of Sexual Satisfaction; MENQOL: Menopause-Specific Quality of Life Questionnaire; MFSQ: McCoy Female Sexuality Questionnaire; MRS: Menopause Rating Scale; PQ: Partnership Questionnaire; SAQ: Sexual Activity Questionnaire; SDS: Sexual Desire Scale; SBQ: Sexual Behavior Questionnaire; SDQ: Sexual Desire Questionnaire; SHI: Sexual History Inventory; SIDI: Sexual Interest/Desire Inventory; SII: Sexual Interaction Inventory; PSI: Problem Solving Inventor; RBI: Relationship Belief Inventory. *Interventions*: EI: Educational Intervention; ST: Sexual Therapy; SST: Sexual Skills Training; OP: Other Psychotherapies *Others*: *M:* mean; *SD:* standard deviation; n.s.: non-significant; n.a.: not applicable.

### Overall effect of psychological interventions

Findings were grouped into 4 categories according to the intervention specificities: (1) Studies assessing the effect of educational interventions on the sexual health of older adults; (2) Studies exploring the effect of sexual therapy and sexual skills training in older age; (3) Studies evaluating the effect of cognitive-behavioral interventions on older adults’ sexual health; and (4) studies investigating the effect of other therapeutical approaches to promote sexual health in older age. Below we discuss results separately by category, namely, educational interventions, sexual therapy and sexual skills training, cognitive-behavioral therapies, and other psychotherapies.

### Educational interventions

Adams et al. (1990) explored the effect of a small-group sex education intervention on sexual knowledge and attitudes. Two 90-min workshops centered on sexuality in older age were conducted with 10 participants. Sessions aimed at discussing myths and stereotypes about sexuality in older age and transmitting information regarding physiological and psychological sexual changes that occur as age progresses and the benefits of maintaining an active sexual life in older age. Pretest and posttest scores on ASKAS revealed the absence of difference before and after the intervention (*p* > .05). The authors added that participants expressed not being interested in changing their attitudes concerning sex-related issues.

By implementing the PLISSIT model of education (Annon, 1976), Goldman and Carroll (1990) explored the effect of a 4-h workshop of sexuality in older age as an adjunct in the treatment of erectile dysfunction. Twenty heterosexual couples where the male partner had a diagnosis of secondary erectile dysfunction were randomly assigned to either a control or experimental group. The workshop was developed aiming at achieving the goals of increasing knowledge about sexuality in older age, promoting self-acceptance, increasing comfort in addressing sexuality-related issues, and promoting satisfaction with own’s sexual expression. Analytical analysis on the comparison of pretest and posttest scores showed that aging sexuality knowledge increased in the experimental group after the intervention [*t*(39) = −4.06, *p* <.001]. No statistically significant changes were observed in sexual attitudes [*t*(39) = 1.37, *p* >.05]. Sexual satisfaction also increased in the experimental group [*t*(38) = 3.63, *p <*.01]. In contrast, frequency of sexual behavior did not change in either the experimental or control group.

Steinke (1988) also aimed to analyze the effect of an educational program on sexual knowledge, attitudes, and satisfaction, and on the frequency of sexual behavior. Twenty-four participants (8 men and 16 women) completed the program which consisted of four 60-m group sessions. The program addressed not only sexual changes with aging and chronic diseases, and issues related to sexual expression in long-term care facilities, but also explored the link between ageism and sexuality. Results indicated that the program had a positive effect on the sexual knowledge for the experimental group [*t*(11) = 2.91, *p <*.05] but had no effect on the frequency of sexual behavior [*t*(10) = −.94, *p* >.05] or sexual satisfaction [*t*(11) = 1.08, *p* >.05].

White and Catania (1982) developed a psychoeducational intervention focused on demystifying erroneous beliefs about sexuality in older age and fostering an accepting environment for sexuality in later years. The lectures covered material on normative changes in sex and aging, the benefits of sex in old age, social barriers to sexual expression and intimacy, and other biopsychosocial factors related to sex and aging (e.g. the role of medical conditions, medications, psychological and relational factors). The program was implemented with 30 older adults, their family members and nursing practitioners. For the purpose of the current study, we will exclusively examine findings regarding older adults. Results revealed significant differences in the experimental group compared with the control group after completing the intervention on sexual knowledge [*F*(2,27) = 32.66, *p* < .001, *R*^2^ = .71], sexual attitudes [*F*(2,27) = 23.33, *p* < .001, *R*^2^ = .63], sexual satisfaction [*F*(2,27) =17.92, *p* < .001, *R*^2 =^ .57], frequency of masturbation [*F*(2,27) = 5.43, *p* < .01, *R*^2 =^ .29] and frequency of sexual intercourse [*F*(2,27) = 12.41, *p* < .001, *R*^2 =^ .48].

Nazarpour et al. (2016) conducted a randomized controlled trial to test the effect of a 2-h education session on the sexual function of postmenopausal women. Forty-eight postmenopausal women from the intervention group completed the program. The program comprised group educational sessions on various aspects of sexual function, the promotion of more positive attitudes about sex following menopause, and the introduction of lubricants to alleviate vulvovaginal pain caused by dryness. Results indicated that participants from the intervention group presented an improvement in overall sexual function (*p* <.05), arousal (*p* <.01), and pain dimension (*p* <.01) when compared to participants allocated to the control group after the implementation of the program.

### Sexual therapy and sexual skills training

Rowland and Haynes (1978) tested the effect of a group sexual therapy program for older couples. The program comprised three phases: the pretreatment phase, the educational phase, and the marital and sexual skills training phase. The pretreatment phase aimed at assessing behavioral changes in response to the initial interview and treatment anticipation. The educational phase provided information about human sexual function, both in general and specifically in the case of older age. The third phase comprised exercises aiming at improving communication within the couple and sexual techniques (e.g. kissing, caressing, genital stimulation). Ten heterosexual couples completed the intervention program. Findings indicated that sexual satisfaction increased over time [*F*(3, 21) = 7.90, *p* < .01] in the experimental group. In regard to sexual behavior, only female oral and genital stimulation changed significantly over time in the experimental group [*F*(3, 21) = 3.71, *p* < .05].

### Cognitive-behavioral therapies

In a controlled trial, Trudel et al. (2008) tested the effect of a marital and sexual wellness program on couples entering retirement. The program applied cognitive-behavioral strategies to promote sexual and marital functioning. Basic communication training, cognitive restructuring and problem-solving training were amongst the techniques used to improve couples’ communication. The sexual component of the program comprised sexual education, sensate focus exercises, sexual fantasies training, as well as cognitive restructuring. When testing the group x time effects, findings indicated that all tested sexual dimensions increased solely for the group that underwent intervention. Namely, sexual functioning [*F*(1, 80) = 9.98, *p* < .01; *F*(12, 69) = 6.17, *p* < .001], sexual desire [*F*(2, 79) = 10.79, *p* < .001], relational and behavioral dimensions of sexual desire [*F*(2, 79) = 25.08, *p* < .001], and the individual/cognitive dimensions of sexual desire [*F*(1, 80) = 55.82, *p* < .001] all changed in the expected direction in the experimental group after completing the intervention. Likewise, the experimental group presented an increase in knowledge about aging sexuality, with sexual attitudes being more permissive after completing the program [*F*(3, 78) = 12.38, *p* < .001].

Alder et al. (2006) also tested the effect of a cognitive-behavioral group intervention for climacteric symptoms. Psychoeducation, group discussions and coping skills training combined with breathing exercises were included in the program.

Pretest and posttest comparisons showed a significant increasement in sexual well-being [*F*(1, 29) = 6.2, *p* < 0.05].

A randomized controlled trial on the efficacy of cognitive-behavioral therapy for menopausal and postmenopausal women was conducted by Green et al. (2019) The program consisted in testing the CBT-Meno program (Green et al., 2012), which focused on reducing a wide range of menopause-related symptomatology (e.g. vasomotor, psychopathology, sleep difficulties and urogenital and sexual complaints). Results indicated that participants in the experimental group reported improvements in sexual interest [*F(*1, 67) = 5.20, *p* < .05] over one week. However, no significant changes were found between experimental and waitlist groups in sexual functioning over one month [*F(*1, 52) = .80, *p* > .05].

### Other psychotherapies

Contrasting with the previous trials, Aliabadi et al. (2021) performed a controlled trial aimed at investigating the effect of a mindfulness-based stress reduction program on the quality of life of postmenopausal women. Participants allocated to the intervention group completed a set of exercises, including acceptance of thoughts, learning how to be aware in the present moment, and how to apply mindfulness in everyday life. Results pertaining to the assessment of the sexual dimension of quality of life specific to menopause showed that the clinical sample presented a significant improvement when compared to the control group immediately after the intervention (*p* < .001). Follow-up assessments revealed that the effect of the program persisted after three months (*p* < .01).

The effect of hypnotic relaxation therapy on sexual pleasure and discomfort of sexually active postmenopausal women was tested in a controlled trial (Johnson et al., 2016). Results suggested that a five-weekly session of hypnotic relaxation therapy compared to an active and structured attention control was effective in increasing sexual pleasure [*F*(1, 90) = 14.87, *p* < .001)] and Total SAQ scores [*F*(1, 90) = 7.85, *p* < .01].

A randomized controlled trial was conducted by Ansari et al. (2019) to test the effectiveness of an emotion regulation-based intervention in promoting the sexual satisfaction of postmenopausal women. The program included exercises intended to enhance emotional regulation strategies (e.g. self-regulation). Specifically, participants were guided throughout the sessions to accomplish different emotional regulation skills (e.g. increasing positive emotions; paying conscious attention to feelings without judgment). The first session included a psychoeducational approach to sexuality and menopause, aiming at clarifying the concept of sexual satisfaction and the outcomes of sexual dissatisfaction. Sexuality was also introduced along the program with exercises aiming at promoting more adequate emotional regulation strategies in a sexual context. By comparing the pretest and posttest scores of the experimental and control groups, results suggested that sexual satisfaction only increased for the group that participated in the program (*p* < .05).

## Discussion

The present review aimed to examine the existing evidence on the efficacy of psychological sexual health interventions in older age. The identification of evidence-based interventions indicated that even though there were multiple programs addressing sexual health in older age, they were not complemented by the empirical validation of their effectiveness. Hence, only 12 studies were included in this review.

The studies that were identified in this review implemented different interventional strategies to promote sexual health in older age, that not only aimed at conveying information and promoting more accepting attitudes toward sexuality and aging, but also providing different psychological, sexual, and relational competencies. Even though one of the interventions was not effective in promoting change in knowledge about sexuality and sexual attitudes (Adams et al., 1990), educational interventions proved to be effective in promoting knowledge (Goldman & Carroll, 1990; Steinke, 1988; White & Catania, 1982). Two educational interventions improved sexual satisfaction (Goldman & Carroll, 1990; White & Catania, 1982), one educational approach promoted more permissive attitudes regarding sexuality in older age as well as an increase in frequency of sexual behavior (White & Catania, 1982) and another educational intervention promoted positive change in female sexual function (Nazarpour et al., 2016).

Cognitive-behavioral interventions were also proven to be effective. Moreover, CBT not only promoted knowledge and more tolerant attitudes concerning sexuality and aging (Alder et al., 2006; Trudel et al., 2008), but also had a positive effect on sexual functioning (Trudel et al., 2008) and sexual well-being (Alder et al., 2006). Findings from the two randomized controlled trials that implemented the same interventional approach are consistent with the aforementioned results, indicating that CBT promoted a positive change in sexual interest (Green et al., 2019) and sexual satisfaction (Ansari et al., 2019).

Only one study applied the principles of sexual therapy in combination with sexual skills training. The same applies to third-wave cognitive behavioral therapies and psychoanalytic approaches. Results suggested that a group program that combined sexual therapy with sexual skills training was effective in promoting sexual satisfaction and increasing the frequency of female sexual activities over time (Rowland & Haynes, 1978). A mindfulness-based stress reduction program also was effective in increasing the sexual quality of life for postmenopausal women (Aliabadi et al., 2021). Similarly, a hypnotic relaxation program also enhanced sexual pleasure in a sample of postmenopausal women (Johnson et al., 2016).

Though research indicates that such therapies tend to be helpful for improving sexual health among older age groups, there are still certain gaps that should be addressed. For instance, Green and colleagues (Green et al., 2019) evaluated the differences between control and experimental groups one week and one month after the implementation of the intervention. Results indicated that after one month the differences between the groups were no longer significant. Such findings along with the scarcity of similar assessments in the remaining studies, highlight that further research is still required to determine the long-term effects of these interventions. Moreover, no study aimed at addressing sexual well-being or sexual pleasure. Factors such as stereotypical representations that are mainly invalidating of sexual interest in older age and the lack of knowledge on how to address sexuality with older men and women (Ezhova et al., 2020; Gore-Gorszewska, 2020; Malta, 2020) might have contributed to the dearth of studies addressing sexual health and well-being in later life, thus reducing the availability of effective interventional tools.

Despite the identification of clinical and randomized trials, research on the effectiveness of psychological health therapies in older age is still limited. For instance, study design and sample size should be addressed. It is well established that randomized controlled trials are amongst the best methods for testing the effectiveness of psychological interventions (Haaga & Stiles, 2000). However, only four of the treatment-outcome studies applied a controlled randomized design (Ansari et al., 2019; Green et al., 2019; Nazarpour et al., 2016; White & Catania, 1982). In two of these studies, sample sizes were rather small for conducting a randomized trial (Ansari et al., 2019; White & Catania, 1982). The sample size was also an issue for most of the studies (Alder et al., 2006; Zippan et al., 2020; Rowland & Haynes, 1978; Steinke, 1988). Feasibility studies are fundamental to inform the design of a controlled trial to be performed. In order to have a high probability of detecting significant clinical changes, trial studies are required to have a sample size of, at least, 70 participants (35 for each group) (Teare et al., 2014), which did not occur with most of the studies included in this review. Conducting randomized trials allows determining an intervention as being empirically validated (Chambless et al., 1998), which directly indicates that research is required to establish evidence-based clinical approaches for promoting sexual health in older age.

The assessment of risk of bias revealed that most randomized studies included in this review were rated as having some bias concerns. Bias arising from the randomization sequencing was the most prevailing. Even though results from most studies are not invalidated by the risk of bias, two studies might present contestable results according to the assessment that was conducted (Goldman & Carroll, 1990; Steinke, 1988), indicating that the effectiveness of educational interventions for promoting sexual health in older age might further caress valid research. Interestingly, results reported by Steinke (1988) indicated the absence of effect of a 4-h workshop in positively changing sexual attitudes, suggesting that further research is required to test the potential effect of brief educational approaches in cognitive factors (e.g. attitudes, beliefs).

The absence of studies that included sexually diverse older groups is a noticeable limitation. In parallel, despite older people being one of the fastest-growing groups of internet users (Flynn et al., 2006; Leung et al., 2007), no empirically validated digital intervention directed to promoting sexual health in older age was identified. E-health platforms emerge as potentially powerful tools for promoting sexual health in older age, providing not only accessibility that surpasses mobility constraints, but also mitigates the barriers that older adults face when accessing healthcare professionals (e.g. invalidation of sexual interest, fear of judgment) (Malta et al., 2020; Minichiello et al., 2013). Such a finding reinforces that despite the potential benefits of providing self-care or digital tools to assist older people in managing sexual health concerns, there is still a paucity in the literature.

Finally, a note on the limitations regarding this review that should be acknowledged. The first consists of the exclusion of non-validated interventions. As previously stated, there are multiple programs aiming at promoting sexual health in older age, and this review focuses exclusively on interventions that evaluated their effectiveness. The age criterion allowed the inclusion of samples with different age ranges, including younger participants. Also, the usage of specific search terms directed to sexuality and sexual health might have restricted the obtained results. The non-pre-registration of the review protocol has also is also a recognizable limitation.

Nonetheless, this review consists of a recent effort to systematize empirically validated psychological sexual health interventions in older age. Recognizing the link between sexual health and overall health and well-being in older age (T. J. Flynn & Gow, 2015; Smith et al., 2019; Willie-Tyndale et al., 2021; Wright et al., 2019) and the establishment of sexual health in older age as a global public health priority (Sadana et al., 2018; UN, 2023; WHO, 2015), the present review provides a potentially useful systematization of the current empirically validated psychological sexual health interventions in older age.

## Conclusions

Findings from this review suggest that varied interventional approaches appear to be somewhat effective in promoting sexual health in older age. Educational interventions are the most frequent approach when addressing sexuality and aging, followed by cognitive-behavioral techniques, sexual therapy combined with sexual skills training, mindfulness-based therapies, and a relaxation program. Despite the mixed results and the lack of randomized controlled trials, it is reasonable to consider educational and cognitive-behavioral approaches when aiming at promoting positive changes in sexual health at older age. However, further research must be performed to determine the empirical validity of psychological interventions on sexual health in older age, particularly by implementing robust methods to reduce risk of bias. In parallel, evidence is still required to investigate the effectiveness of these interventional approaches in sexually diverse older groups, to test how these effects stand through time, and to identify the principles and change mechanisms of such interventions.
